# Synthesis and structure of (3a*RS*,10*SR*,10a*SR*)-2-(4-chloro­phen­yl)-5-[(4-methyl­phen­yl)sulfon­yl]-1-oxo-1,2,3,3a,4,5,10,10a-octa­hydro­pyrrolo­[3,4-*b*]carbazole-10-carb­oxy­lic acid with an unknown solvent

**DOI:** 10.1107/S2056989026003762

**Published:** 2026-04-17

**Authors:** Elizaveta D. Yakovleva, Elena A. Sorokina, Victor N. Khrustalev, Mehmet Akkurt, Khudayar I. Hasanov, Narmina A. Guliyeva, Nurlana D. Sadikhova, Menberu Mengesha Woldemariam

**Affiliations:** a RUDN University, 6 Miklukho-Maklaya St., Moscow 117198, Russian Federation; b Zelinsky Institute of Organic Chemistry of RAS, Leninsky Prospect 47, 119991 Moscow, Russian Federation; c Department of Physics, Faculty of Sciences, Erciyes University, 38039 Kayseri, Türkiye; d Azerbaijan Medical University, Scientific Research Centre (SRC), A. Kasumzade St. 14, AZ 1022, Baku, Azerbaijan; e Department of Chemical Engineering, Baku Engineering University, Khirdalan, Hasan Aliyev str. 120, AZ0101 Absheron, Azerbaijan; f Organic Chemistry Department, Baku State University, Z. Khalilov str. 23, AZ 1148, Baku, Azerbaijan; g Department of Physics, Jimma University, Jimma, Ethiopia; University of Aberdeen, United Kingdom

**Keywords:** crystal structure, 2,3-di­hydro-1*H*-pyrrole, cyclo­hexa­ne, pyrrolidine, Hirshfeld surface analysis

## Abstract

The title compound, C_28_H_23_ClN_2_O_5_S, crystallizes with two independent mol­ecules (*A* and *B*) in the asymmetric unit. In the central fused ring systems, the pyrrolidine rings adopt envelope conformations and the cyclo­hexene rings adopt distorted-half chair conformations. In mol­ecules *A* and *B*, the least-squares mean planes of the sixteen-membered central ring systems form dihedral angles of 81.5 (1) and 84.0 (1)°, respectively, with the benzene rings attached to the sulfanyl groups, while they make dihedral angles of 18.6 (1) and 7.7 (1)°, respectively, with the chloro­benzene rings. In the crystal, O—H⋯O hydrogen bonds and weak C—H⋯O and C—H⋯Cl inter­actions link the mol­ecules, forming a three-dimensional network. The contribution of some disordered solvent to the scattering was removed using the SQUEEZE routine [Spek (2015 ▸). *Acta Cryst*. C71, 9–18] in *PLATON*. The solvent contribution was not included in the reported mol­ecular weight and density. A Hirshfeld surface analysis indicates that H⋯H (36.8% for mol­ecule *A*; 29.8% for mol­ecule *B*), O⋯H/H⋯O (22.1% for *A* and 27.3% for *B*), C⋯H/H⋯C (22.1% for *A* and 20.2% for *B*) and Cl⋯H/H⋯Cl (9.7% for *A* and 13.2% for *B*) inter­actions are the most important contributors to the crystal packing.

## Abstract 2

The title compound crystallizes with two independent mol­ecules in the asymmetric unit. In the extended structure, O—H⋯O hydrogen bonds and C—H⋯O and C—H⋯Cl inter­actions link the mol­ecules to form a three-dimensional network. Furthermore, C—H⋯π inter­actions are also observed.

## 1. Chemical context

Oxidative stress is a key factor in the progression of many diseases, including cardiovascular diseases, diabetes, neurological disorders like Alzheimer’s and Parkinson’s, cancer, and inflammatory conditions (Cheresh *et al.*, 2013 ▸). Iso­indole derivatives possess a wide range of biological activities, including anti­oxidant properties, which are relevant to conditions like fibrosis, making the annulated scaffold a promising area for further drug development. Pyrrolo­[4-*b*]carbazole-10-carb­oxy­lic acid belongs to the larger class of carbazole derivatives, which are known for their diverse biological activities with the putative mode of action involving inhibition of oxidative processes (in particular, non-enzymatic glycation, some mechanistic steps of which are oxidation-dependent) (Ibragimova *et al.*, 2024 ▸). In a continuation of our research in this area (Horak *et al.*, 2015 ▸; Polyanskii *et al.*, 2019 ▸; Shelukho *et al.*, 2025 ▸; Zubkov *et al.*, 2016 ▸), we developed an efficient synthetic protocol involving acid-catalysed isomerization in 1,2-di­chloro­ethane with an equimolar amount of hydrogen chloride in dioxane for the aromatization of [4 + 2]-cyclo­addition adducts and we now describe the synthesis and structure of the title compound, C_28_H_23_ClN_2_O_5_S (**I**).
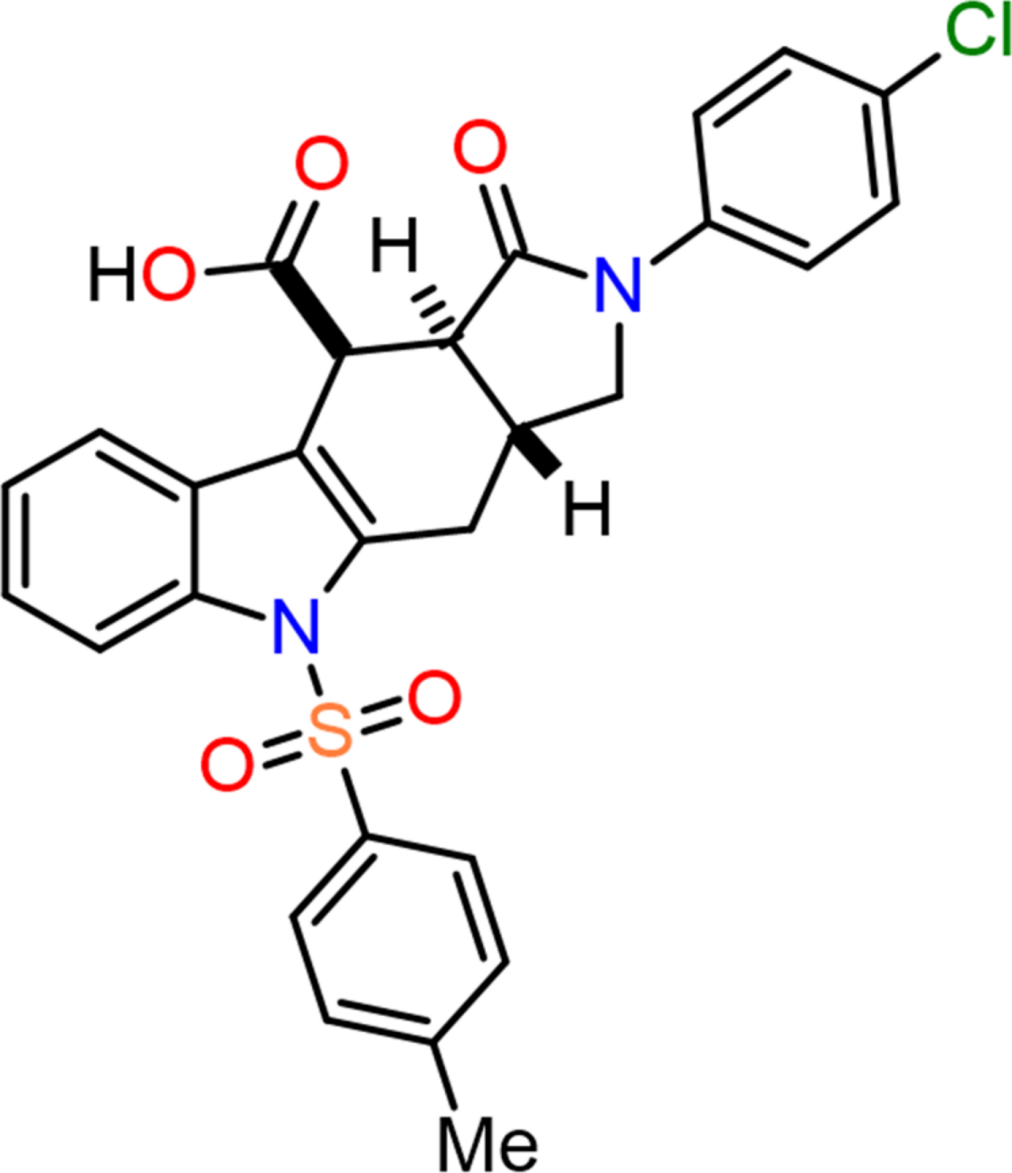


## 2. Structural commentary

**Figure 1:**
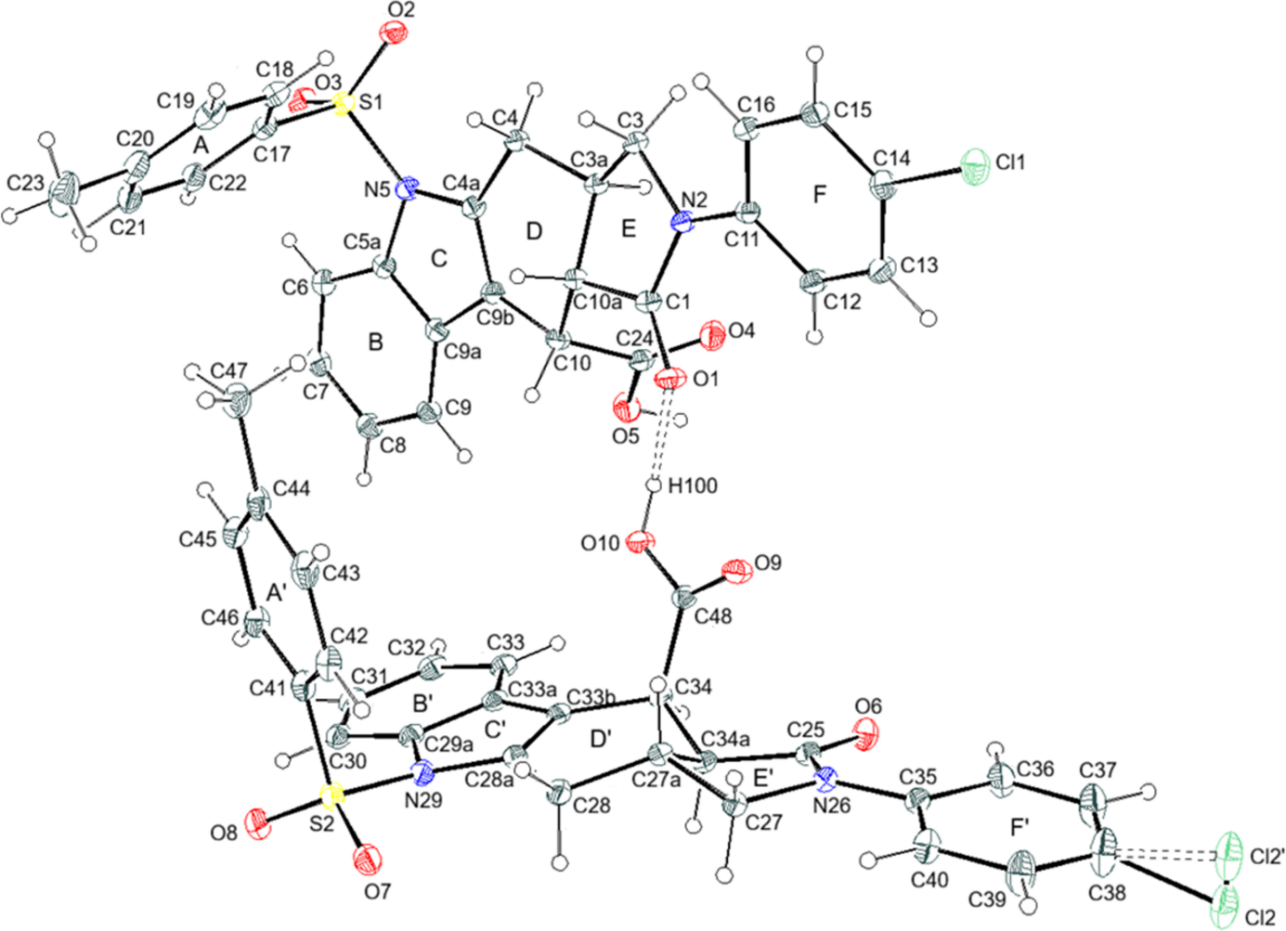
The mol­ecular structure of the two independent mol­ecules (*A* and *B*) of (**I**), showing the atom labelling. Displacement ellipsoids are drawn at the 30% probability level. The dashed line indicates the strong O—H⋯O hydrogen bond.

The title compound (Fig. 1 ▸) crystallizes with two mol­ecules *A* (containing Cl1) and *B* (containing Cl2) in the asymmetric unit in the triclinic space group *P*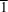. In the central 19-atom fused ring systems, the 2,3-di­hydro-1*H*-pyrrole rings [*A* (*C*): C4*A*/N5/C5*A*/C9*A*/C9*B* and *B* (*C*′): C28*A*/N29/C29*A*/C33*A*/C33*B*] adopt essentially planar conformations (r.m.s deviation = 0.001 Å for both mol­ecules), the cyclo­hexane rings [*A* (*D*): C3*A*/C4/C4*A*/C9*B*/C10/C10*A* and *B* (*D*′): C27*A*/C28/C28*A*/C33*B*/C34/C34*A*] adopt distorted-half chair conformations [Cremer–Pople puckering parameters *Q*_T_ = 0.5442 (15) Å, θ = 52.41 (16)°, φ(2) = 322.5 (2)° and *Q*_T_ = 0.5239 (15) Å, θ = 51.37 (16)°, φ(2) = 321.5 (2)°, respectively] and the pyrrolidine rings are in envelope conformations [*A* (*E*) C1/N2/C3/C3*A*/C10*A*; *Q*_T_ = 0.3618 (15) Å, φ(2) = 108.9 (2)° and *B* (*E*′) C25/N26/C27/C27*A*/C34*A*, *Q*_T_ = 0.3485 (15) Å, φ(2) = 105.4 (2)°]. Each mol­ecule in the arbitrarily chosen asymmetric unit has three stereogenic (chiral) centres (C3*A R*, C10 *S*, C10*A S* and C27*A R*, C34 *S*, C34*A S*) but crystal symmetry generates a racemic mixture.

Overall, the central fused ring systems are roughly planar (r.m.s deviations of 0.177 and 0.191 Å for mol­ecules *A* and *B*, respectively). They form dihedral angles of 81.5 (1) and 84.0 (1)°, respectively, with the benzene rings of the 1-(dioxo-λ^6^-sulfan­yl)-4-methyl­benzene groups [*A* (*A*): C17–C22 and *B* (*A*′): C41–C46], while they make dihedral angles of 18.6 (1) and 7.7 (1)°, respectively, with the benzene rings of the chloro­benzene groups.

## 3. Supra­molecular features and Hirshfeld surface analysis

**Figure 2:**
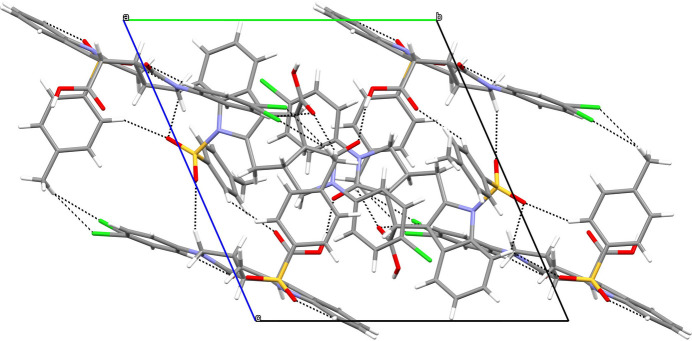
The packing of (**I**), viewed down the *a*-axis direction, showing O—H⋯O, C—H⋯O and C—H⋯Cl hydrogen bonds.

In the crystal, strong O10—H100⋯O1 hydrogen bonds link the mol­ecules into *A* + *B* dimers. The O5—H50 moiety probably forms a hydrogen bond to a disordered solvent mol­ecule. Weak C—H⋯O, C—H⋯Cl and C—H⋯π inter­actions link the dimers, thereby forming a three-dimensional network (Table 1 ▸, Fig. 2 ▸). For further packing figures, see the supporting information

**Figure 3:**
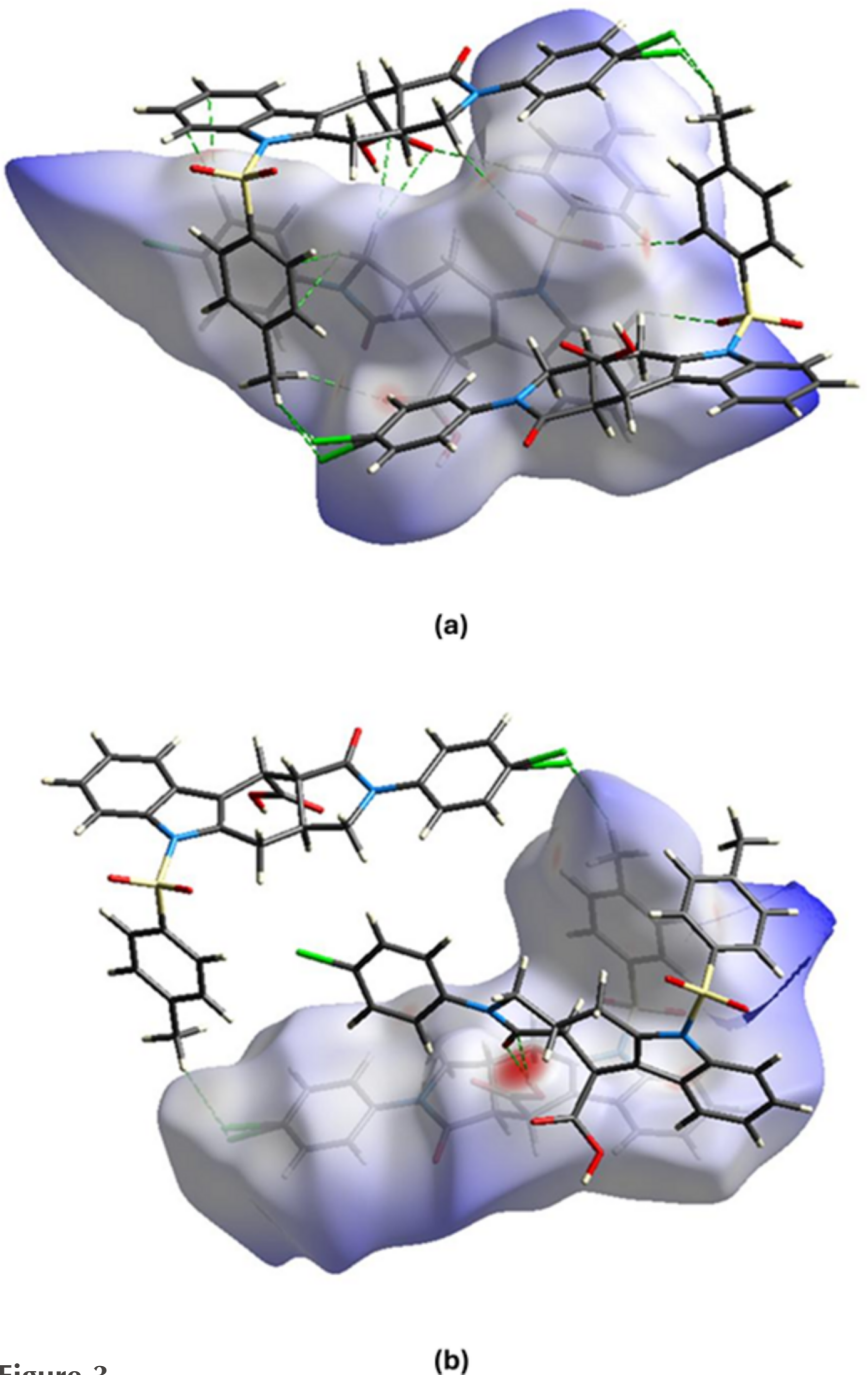
The views of the three-dimensional Hirshfeld surfaces of mol­ecules *A* and *B* of the title compound plotted over *d*_norm_ are shown in (*a*) and (*b*), respectively.

*CrystalExplorer 17.5* (Spackman *et al.*, 2021 ▸) was used to construct Hirshfeld surfaces and generate the related two dimensional fingerprint plots to illustrate the inter­molecular inter­actions for mol­ecules *A* and *B*. The *d*_norm_ mappings of mol­ecules *A* and *B* were conducted in the range −0.74 to +4.50 a.u. and −0.74 to +6.10 a.u., respectively. Bright-red circles on the *d*_norm_ surfaces (Fig. 3 ▸) represent H⋯H, O—H⋯O, C—H⋯O and C—H⋯Cl inter­action zones (Tables 1 ▸ and 2 ▸).

**Figure 4:**
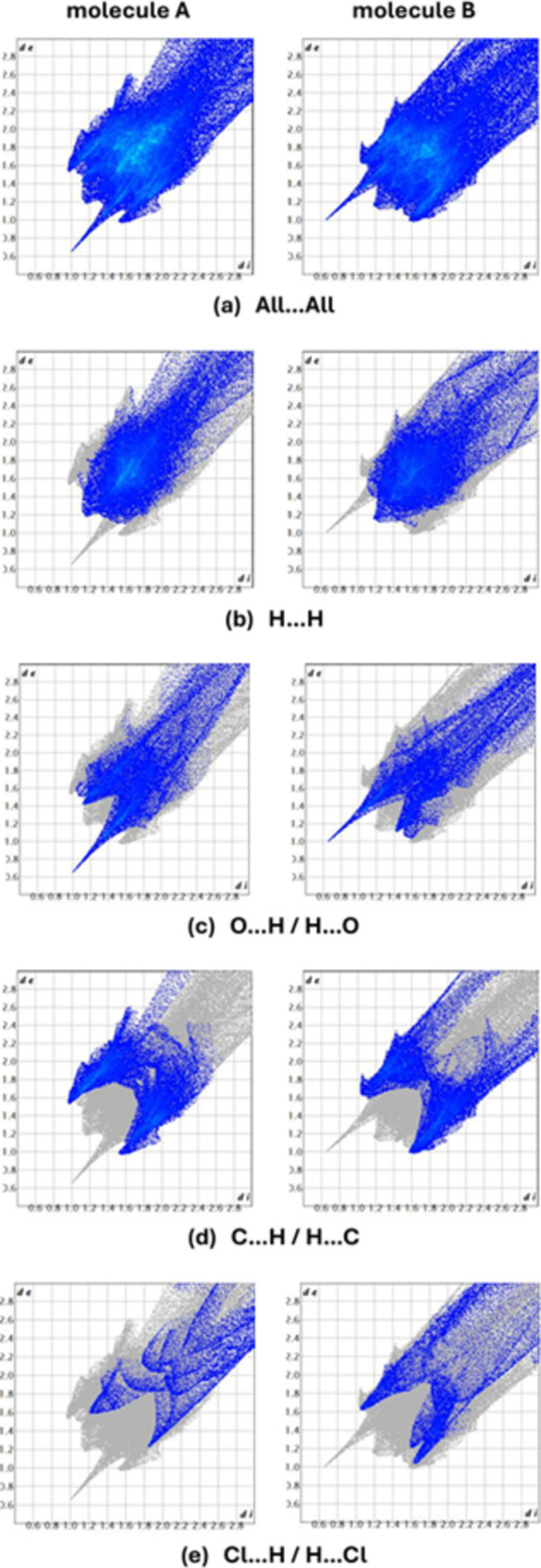
The two-dimensional fingerprint plots of mol­ecules *A* and *B* of the title compound, showing (*a*) all inter­actions, and delineated into (*b*) H⋯H, (*c*) O⋯H/H⋯O, (*d*) C⋯H/H⋯C, and (*e*) Cl⋯H/H⋯Cl inter­actions [*d*_e_ and *d*_i_ represent the distances from a point on the Hirshfeld surface to the nearest atoms outside (external) and inside (inter­nal) the surface, respectively].

Two-dimensional fingerprint plots together with their percentage contributions are shown in Fig. 4 ▸ and Table 2 ▸. The crystal packing is dominated by H⋯H contacts, representing van der Waals inter­actions (36.8% for mol­ecule *A* and 29.8% for mol­ecule *B*), followed by O⋯H/H⋯O (22.1% for *A* and 27.3% for *B*), C⋯H/H⋯C (22.1% for *A* and 20.2% for *B*) and Cl⋯H/H⋯Cl inter­actions (9.7% for *A* and 13.2% for *B*). The other contacts contribute 3.2% or less, and the details of these are provided in Table 2 ▸. The different values for mol­ecules *A* and *B* in the table are due to the fact that the mol­ecular environments of these mol­ecules within the crystal are not exactly identical, including the disordered solvent mol­ecules.

## 4. Database survey

A search of the Cambridge Structural Database (CSD, version 6.00, update April 2025; Groom *et al.*, 2016 ▸) for the octa­hydro-1*H*-isoindol-1-one unit gave 469 hits. The seven compounds closely related to (**I**) have CSD refcodes EHURIM (Yakovleva *et al.* 2025 ▸), MIYNAN (Mammadova *et al.*, 2023 ▸), ANAMUZ (Mariaule *et al.*, 2016 ▸), BAFYAL (Zhong *et al.*, 2017 ▸), NAMROK (Chou & Wu, 2012 ▸), TODKEF (Elliott & Booker-Milburn, 2019 ▸) and YOPXIL (Paddon-Row *et al.*, 2009 ▸).

In the crystal of EHURIM, the mol­ecules are connected by C—H⋯O hydrogen bonds, forming layers lying parallel to the (101) plane. Furthermore, the mol­ecules form layers parallel to the (10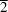) plane by way of C—H⋯π inter­actions. In MIYNAN, mol­ecules are connected by pairwise C—H⋯O hydrogen bonds, forming dimers with an 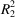
(8) motif. These dimers form a three-dimensional network through O—H⋯O, O—H⋯S and C—H⋯O hydrogen bonds with each other directly and through solvent mol­ecules. In addition, weak π–π stacking inter­actions are observed. In the structure of ANAMUZ, the mol­ecules are linked by C—H⋯O and O—H⋯O hydrogen bonds, forming a three-dimensional network. Weak π–π inter­actions are also observed. In BAFYAL, the mol­ecules are linked by C—H⋯O inter­actions, forming layers lying parallel to the (002) plane and π–π inter­actions are also present. In NAMROK, pairs of mol­ecules are linked by C—H⋯O inter­actions but π–π and C—H⋯π inter­actions are not observed. In TODKEF, the mol­ecules are linked by C—H⋯O and O—H⋯O hydrogen bonds, forming a three-dimensional network; C—H⋯π inter­actions are also observed. In YOPXIL, the mol­ecules are linked by C—H⋯O hydrogen bonds, forming chains along the *b*-axis direction. No π–π or C—H⋯π inter­actions are observed.

## 5. Synthesis and crystallization

An equimolar amount of HCl in dioxane (5.0 mol L^−1^; 0.250 mmol, 0.0045 mL) was added to a suspension of (3a*RS*,9b*SR*,10*RS*,10a*SR*)-2-(4-chloro­phen­yl)-5-[(4-methyl­phen­yl)sulfon­yl]-1-oxo-1,2,3,3a,5,9b,10,10a-octa­hydro­pyrrolo­[3,4-*b*]carbazole-10-carb­oxy­lic acid (0.250 mmol, 0.13 g) in DCE (10 mL). The resulting mixture was stirred at r.t. for 24 h. The resulting precipitate was filtered off, washed with diethyl ether (5 mL), and air-dried to afford the target product as white powder (0.21 mmol, 87%). Single crystals suitable for X-ray diffraction were obtained by slow evaporation of a mixture of ethanol and DMF. Yield 87%, 0.11 g; m.p. 487–491 K. IR (KBr): 3055 (OH), 1735 (CO_2_), 1648 (N—C=O). ^1^H NMR (600 MHz, DMSO-*d*_6_, 298 K) δ 12.80 (*br.s.*, 1H, H CO_2_H), 8.04 (*d*, *J* = 7.6 Hz, 1H, H Ar), 7.82 (*d*, *J* = 8.6 Hz, 2H, H Ar), 7.75 (*d*, *J* = 7.6 Hz, 1H, H Ar), 7.72 (*d*, *J* = 9.1 Hz, 2H, H Ar), 7.46 (*d*, *J* = 9.1 Hz, 2H, H Ar), 7.37 (*d*, *J* = 8.6 Hz, 1H, H Ar), 7.32 (*t*, *J* = 7.6 Hz, 1H, H Ar), 7.28 (*t*, *J* = 7.6 Hz, 1H, H Ar), 4.13 (*d*, *J* = 4.5 Hz, 1H, H-10), 4.09 (*dd*, *J* = 8.6, 7.6 Hz, 1H, H-3A), 3.81 (*dd*, *J* = 10.6, 9.1 Hz, 1H, H-3B), 3.58 (*dd*, *J* = 16.6, 4.5 Hz, 1H, H-10a), 3.27–3.19 (*m*, 1H, H-3a), 3.01 (*dd*, *J* = 16.1, 11.6 Hz, 1H, H-3A), 3.01 (*dd*, *J* = 13.1, 5.1 Hz, 1H, H-3B), 2.32 (*s*, 3H, CH_3_) ppm. ^13^C NMR (150.9 MHz, DMSO-*d*_6_, 298 K) δ 172.9, 172.6, 146.1, 139.2, 136.8, 136.0, 135.2, 130.9 (2C), 129.2 (2C), 129.0, 128.0, 127.0 (2C), 125.1, 124.0, 121.0 (2C), 120.6, 116.5, 114.3, 51.8, 47.1, 37.3, 32.2, 28.3, 21.6 ppm. MS (ESI): *m/z* = 535 [*M* + H, ^35^Cl]^+^, 537 [*M* + H, ^37^Cl]^+^. Analysis calculated for C_28_H_23_ClN_2_O_5_S: C 62.86, H 4.33, N 5.24, S 5.99; found: C 62.51, H 4.22, N 5.52, S 6.12.

## 6. Refinement

Crystal data, data collection and structure refinement details are summarized in Table 3 ▸. All C-bound H atoms were positioned geometrically (C—H = 0.95 and 1.00 Å) and included as riding contributions with isotropic displacement parameters fixed at 1.2*U*_eq_(C) (1.5 for methyl groups). The H atoms of the OH groups were found from difference-Fourier maps and refined freely. Atom Cl2 of mol­ecule *B* exhibits disorder over two positions in the ratio 0.60:0.40. The residual electron density was difficult to model and therefore the SQUEEZE routine in *PLATON* (Spek, 2020 ▸) was used to remove the contribution of the electron density in the solvent region from the intensity data and the solvent-free model was employed for the final refinement. The cavity of volume *ca*. 561 Å^3^ (*ca* 19.8% of the unit-cell volume) contains approximately 163 electrons. A suitable solvent with this electron number may be about four *N*,*N*-di­methyl­formamide mol­ecules per unit cell. The solvent formula mass was not taken into account when calculating the crystal density, *etc*.

## Supplementary Material

Crystal structure: contains datablock(s) I. DOI: 10.1107/S2056989026003762/hb8183sup1.cif

Structure factors: contains datablock(s) I. DOI: 10.1107/S2056989026003762/hb8183Isup2.hkl

Supplementary materials. DOI: 10.1107/S2056989026003762/hb8183sup3.docx

Supporting information file. DOI: 10.1107/S2056989026003762/hb8183Isup4.cml

CCDC reference: 2545066

Additional supporting information:  crystallographic information; 3D view; checkCIF report

## Figures and Tables

**Table 1:** Hydrogen-bond geometry (Å, °) *Cg*2, *Cg*4, *Cg*6, *Cg*11 and *Cg*12 are the centroids of the C28*A*/N29/C29*A*/C33*A*/C33*B*, C29*A*/C30–C33/C33*A*, C41–C46, C11–C16 and C17–C22 rings, respectively.

*D*—H⋯*A*	*D*—H	H⋯*A*	*D*⋯*A*	*D*—H⋯*A*
O10—H100⋯O1	0.90 (3)	1.74 (3)	2.6219 (14)	166 (2)
C27—H27*A*⋯O2^i^	0.99	2.48	3.3125 (18)	141
C39—H39⋯O4^ii^	0.95	2.50	3.380 (3)	155
C42—H42⋯O3^ii^	0.95	2.49	3.324 (2)	147
C45—H45⋯Cl2^iii^	0.95	2.80	3.599 (2)	143
C47—H47*A*⋯O4^i^	0.98	2.48	3.338 (2)	146
C3—H3*A*⋯*Cg*6^i^	0.99	2.86	3.7276 (16)	147
C13—H13⋯*Cg*12^i^	0.95	2.60	3.5017 (18)	158
C15—H15⋯*Cg*4^i^	0.95	2.60	3.4274 (16)	145
C34*A*—H34*A*⋯*Cg*2^iv^	1.00	2.58	3.5252 (14)	157
C45—H45⋯*Cg*11^i^	0.95	2.98	3.3309 (17)	103

Symmetry codes: (i) 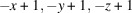; (ii) 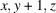; (iii) 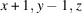; (iv) 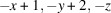.

**Table 2:** Percentage contributions of inter­atomic contacts to the Hirshfeld surfaces for mol­ecules *A* and *B* of the title compound

Contact	Mol­ecule *A*	Mol­ecule *B*
H⋯H	36.8	29.8
O⋯H/H⋯O	22.1	27.3
C⋯H/H⋯C	22.1	20.2
Cl⋯H/H⋯Cl	9.7	13.2
Cl⋯C/C⋯C	3.2	0.1
C⋯C	2.3	1.6
N⋯H/H⋯N	1.5	2.0
Cl⋯Cl	0.8	0.8
O⋯O	0.6	3.7
O⋯C/C⋯O	0.5	0.5
O⋯N/N⋯O	0.3	0.3
N⋯C/C⋯N	–	0.4

**Table 3:** Experimental details

Crystal data	
Chemical formula	C_28_H_23_ClN_2_O_5_S
*M*_r_	534.99
Crystal system, space group	Triclinic, *P* 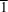
Temperature (K)	100
*a*, *b*, *c* (Å)	14.4661 (2), 14.6841 (2), 15.0214 (2)
α, β, γ (°)	66.157 (1), 86.622 (1), 76.512 (1)
*V* (Å^3^)	2835.81 (7)
*Z*	4
Radiation type	Cu *K*α
μ (mm^−1^)	2.20
Crystal size (mm)	0.23 × 0.14 × 0.08

Data collection	
Diffractometer	Rigaku XtaLAB Synergy-S, HyPix-6000HE area-detector
Absorption correction	Multi-scan (*CrysAlis PRO*; Rigaku OD, 2025 ▸)
*T*_min_, *T*_max_	0.624, 0.838
No. of measured, independent and observed [*I* > 2σ(*I*)] reflections	74492, 12258, 11350
*R*_int_	0.034
(sin θ/λ)_max_ (Å^−1^)	0.639

Refinement	
*R*[*F*^2^ > 2σ(*F*^2^)], *wR*(*F*^2^), *S*	0.041, 0.115, 1.07
No. of reflections	12258
No. of parameters	686
H-atom treatment	H atoms treated by a mixture of independent and constrained refinement
Δρ_max_, Δρ_min_ (e Å^−3^)	0.61, −0.52

Computer programs: *CrysAlis PRO* (Rigaku OD, 2025 ▸), *SHELXT2016/6* (Sheldrick, 2015*a* ▸), *SHELXL2016/6* (Sheldrick, 2015*b* ▸, *ORTEP-3 for Windows* (Farrugia, 2012 ▸) and *PLATON* (Spek, 2020 ▸).

## supplementary crystallographic information

### (3aRS,10SR,10aSR)-2-(4-Chlorophenyl)-5-[(4-methylphenyl)sulfonyl]-1-oxo-1,2,3,3a,4,5,10,10a-octahydropyrrolo[3,4-b]carbazole-10-carboxylic acid Crystal data

**Table:**

C_28_H_23_ClN_2_O_5_S	*Z* = 4
*M**_r_* = 534.99	*F*(000) = 1112
Triclinic, *P*1	*D*_x_ = 1.253 Mg m^−^^3^
*a* = 14.4661 (2) Å	Cu *K*α radiation, λ = 1.54184 Å
*b* = 14.6841 (2) Å	Cell parameters from 44592 reflections
*c* = 15.0214 (2) Å	θ = 3.1–79.7°
α = 66.157 (1)°	µ = 2.20 mm^−^^1^
β = 86.622 (1)°	*T* = 100 K
γ = 76.512 (1)°	Prism, colourless
*V* = 2835.81 (7) Å^3^	0.23 × 0.14 × 0.08 mm

### (3aRS,10SR,10aSR)-2-(4-Chlorophenyl)-5-[(4-methylphenyl)sulfonyl]-1-oxo-1,2,3,3a,4,5,10,10a-octahydropyrrolo[3,4-b]carbazole-10-carboxylic acid Data collection

**Table:**

Rigaku XtaLAB Synergy-S, HyPix-6000HE area-detector diffractometer	11350 reflections with *I* > 2σ(*I*)
Radiation source: micro-focus sealed X-ray tube	*R*_int_ = 0.034
φ and ω scans	θ_max_ = 80.0°, θ_min_ = 3.1°
Absorption correction: multi-scan (CrysAlisPro; Rigaku OD, 2025)	*h* = −18→18
*T*_min_ = 0.624, *T*_max_ = 0.838	*k* = −17→18
74492 measured reflections	*l* = −19→19
12258 independent reflections	

### (3aRS,10SR,10aSR)-2-(4-Chlorophenyl)-5-[(4-methylphenyl)sulfonyl]-1-oxo-1,2,3,3a,4,5,10,10a-octahydropyrrolo[3,4-b]carbazole-10-carboxylic acid Refinement

**Table:**

Refinement on *F*^2^	Primary atom site location: difference Fourier map
Least-squares matrix: full	Secondary atom site location: difference Fourier map
*R*[*F*^2^ > 2σ(*F*^2^)] = 0.041	Hydrogen site location: mixed
*wR*(*F*^2^) = 0.115	H atoms treated by a mixture of independent and constrained refinement
*S* = 1.07	*w* = 1/[σ^2^(*F*_o_^2^) + (0.0691*P*)^2^ + 0.879*P*] where *P* = (*F*_o_^2^ + 2*F*_c_^2^)/3
12258 reflections	(Δ/σ)_max_ = 0.001
686 parameters	Δρ_max_ = 0.61 e Å^−^^3^
0 restraints	Δρ_min_ = −0.52 e Å^−^^3^

### (3aRS,10SR,10aSR)-2-(4-Chlorophenyl)-5-[(4-methylphenyl)sulfonyl]-1-oxo-1,2,3,3a,4,5,10,10a-octahydropyrrolo[3,4-b]carbazole-10-carboxylic acid Special details

**Table:**

Geometry. All esds (except the esd in the dihedral angle between two l.s. planes) are estimated using the full covariance matrix. The cell esds are taken into account individually in the estimation of esds in distances, angles and torsion angles; correlations between esds in cell parameters are only used when they are defined by crystal symmetry. An approximate (isotropic) treatment of cell esds is used for estimating esds involving l.s. planes.

### (3aRS,10SR,10aSR)-2-(4-Chlorophenyl)-5-[(4-methylphenyl)sulfonyl]-1-oxo-1,2,3,3a,4,5,10,10a-octahydropyrrolo[3,4-b]carbazole-10-carboxylic acid Fractional atomic coordinates and isotropic or equivalent isotropic displacement parameters (Å^2^)

**Table:**

	*x*	*y*	*z*	*U*_iso_*/*U*_eq_	Occ. (<1)
Cl1	0.19060 (3)	0.64209 (3)	0.79835 (3)	0.03674 (9)	
S1	0.65573 (2)	0.03992 (2)	0.44576 (2)	0.02786 (8)	
O1	0.39800 (8)	0.57838 (7)	0.40647 (7)	0.0332 (2)	
O2	0.60157 (8)	0.00763 (7)	0.53066 (7)	0.0324 (2)	
O3	0.68382 (8)	−0.02374 (8)	0.39334 (8)	0.0360 (2)	
O4	0.29450 (8)	0.47543 (9)	0.30948 (8)	0.0398 (3)	
O5	0.36563 (8)	0.46953 (10)	0.17543 (8)	0.0410 (3)	
C1	0.40762 (10)	0.48523 (10)	0.45124 (9)	0.0262 (3)	
N2	0.37542 (8)	0.43919 (8)	0.54193 (8)	0.0249 (2)	
C3	0.40760 (10)	0.32626 (10)	0.57978 (9)	0.0250 (2)	
H3A	0.357475	0.292380	0.616858	0.030*	
H3B	0.466032	0.301174	0.621907	0.030*	
C3A	0.42685 (9)	0.30851 (10)	0.48624 (9)	0.0247 (2)	
H3C	0.365084	0.311745	0.457218	0.030*	
C4	0.49735 (10)	0.21070 (10)	0.49343 (9)	0.0269 (3)	
H4A	0.550922	0.194792	0.539400	0.032*	
H4B	0.465744	0.152628	0.517313	0.032*	
C4A	0.53330 (9)	0.22777 (10)	0.39307 (10)	0.0258 (3)	
N5	0.58991 (8)	0.15020 (9)	0.36627 (8)	0.0271 (2)	
C5A	0.61316 (10)	0.19760 (11)	0.26700 (10)	0.0277 (3)	
C6	0.66880 (10)	0.15718 (11)	0.20698 (11)	0.0307 (3)	
H6	0.700781	0.087030	0.230682	0.037*	
C7	0.67567 (11)	0.22343 (12)	0.11117 (11)	0.0340 (3)	
H7	0.712661	0.197793	0.068371	0.041*	
C8	0.62951 (11)	0.32708 (12)	0.07591 (10)	0.0336 (3)	
H8	0.635703	0.370457	0.009901	0.040*	
C9	0.57492 (10)	0.36715 (11)	0.13614 (10)	0.0303 (3)	
H9	0.543854	0.437577	0.112286	0.036*	
C9A	0.56654 (10)	0.30145 (11)	0.23305 (10)	0.0270 (3)	
C9B	0.51768 (9)	0.31837 (10)	0.31320 (10)	0.0262 (3)	
C10	0.46518 (10)	0.41939 (10)	0.31519 (9)	0.0268 (3)	
H10	0.502916	0.471666	0.280007	0.032*	
C10A	0.46350 (10)	0.40284 (10)	0.42184 (9)	0.0250 (2)	
H10A	0.531031	0.389646	0.443929	0.030*	
C11	0.33272 (9)	0.48988 (10)	0.60190 (9)	0.0249 (2)	
C12	0.27819 (10)	0.59029 (10)	0.56196 (10)	0.0290 (3)	
H12	0.270364	0.626545	0.493268	0.035*	
C13	0.23549 (11)	0.63697 (11)	0.62267 (11)	0.0311 (3)	
H13	0.199022	0.705470	0.595624	0.037*	
C14	0.24614 (10)	0.58343 (11)	0.72296 (10)	0.0294 (3)	
C15	0.30053 (10)	0.48442 (11)	0.76343 (10)	0.0288 (3)	
H15	0.308150	0.448577	0.832178	0.035*	
C16	0.34396 (10)	0.43771 (10)	0.70287 (10)	0.0271 (3)	
H16	0.381572	0.369752	0.730410	0.033*	
C17	0.75668 (10)	0.07144 (10)	0.47492 (11)	0.0298 (3)	
C18	0.75855 (11)	0.08893 (11)	0.55916 (11)	0.0336 (3)	
H18	0.706120	0.083528	0.600654	0.040*	
C19	0.83802 (13)	0.11435 (12)	0.58166 (13)	0.0413 (4)	
H19	0.839794	0.126222	0.639122	0.050*	
C20	0.91530 (12)	0.12275 (12)	0.52129 (15)	0.0442 (4)	
C21	0.91189 (12)	0.10370 (13)	0.43803 (14)	0.0435 (4)	
H21	0.964626	0.108246	0.396931	0.052*	
C22	0.83348 (11)	0.07831 (12)	0.41386 (12)	0.0360 (3)	
H22	0.831986	0.065802	0.356719	0.043*	
C23	0.99969 (15)	0.15524 (17)	0.5438 (2)	0.0640 (6)	
H23A	0.982954	0.229061	0.526924	0.096*	
H23B	1.053491	0.139064	0.505761	0.096*	
H23C	1.017348	0.118666	0.613440	0.096*	
C24	0.36561 (10)	0.45772 (10)	0.26733 (10)	0.0288 (3)	
Cl2	0.02936 (8)	1.39438 (9)	0.29072 (9)	0.0501 (2)	0.6
Cl2'	0.02441 (12)	1.35489 (15)	0.33256 (14)	0.0531 (4)	0.4
S2	0.79363 (2)	0.86805 (3)	0.13611 (3)	0.03020 (9)	
O6	0.23818 (7)	1.01084 (8)	0.14754 (8)	0.0345 (2)	
O7	0.77753 (8)	0.96389 (8)	0.14459 (9)	0.0372 (2)	
O8	0.86107 (7)	0.84581 (9)	0.07033 (8)	0.0364 (2)	
O9	0.36258 (8)	0.81137 (7)	0.29406 (7)	0.0313 (2)	
O10	0.42528 (8)	0.69988 (7)	0.22780 (7)	0.0303 (2)	
C25	0.31367 (10)	1.02023 (10)	0.17024 (9)	0.0263 (3)	
N26	0.32700 (8)	1.07686 (8)	0.22073 (8)	0.0261 (2)	
C27	0.42908 (9)	1.07401 (10)	0.23176 (10)	0.0264 (3)	
H27A	0.444828	1.067500	0.297636	0.032*	
H27B	0.446545	1.135940	0.182450	0.032*	
C27A	0.47855 (9)	0.97835 (10)	0.21541 (9)	0.0247 (2)	
H27C	0.478643	0.917365	0.277731	0.030*	
C28	0.57987 (9)	0.97121 (10)	0.18000 (10)	0.0272 (3)	
H28A	0.586388	1.038269	0.129965	0.033*	
H28B	0.625503	0.951015	0.234946	0.033*	
C28A	0.59967 (9)	0.89201 (10)	0.13744 (9)	0.0253 (2)	
N29	0.68874 (8)	0.85985 (9)	0.10129 (8)	0.0272 (2)	
C29A	0.67850 (10)	0.78957 (10)	0.06158 (9)	0.0270 (3)	
C30	0.74301 (11)	0.73914 (11)	0.01419 (10)	0.0308 (3)	
H30	0.807185	0.745506	0.007344	0.037*	
C31	0.70968 (11)	0.67942 (11)	−0.02247 (10)	0.0335 (3)	
H31	0.752224	0.643441	−0.054202	0.040*	
C32	0.61480 (11)	0.67084 (11)	−0.01385 (10)	0.0329 (3)	
H32	0.593965	0.629967	−0.040414	0.040*	
C33	0.55086 (11)	0.72135 (10)	0.03307 (10)	0.0292 (3)	
H33	0.486604	0.715261	0.039083	0.035*	
C33A	0.58316 (10)	0.78154 (10)	0.07136 (9)	0.0260 (3)	
C33B	0.53545 (9)	0.84680 (9)	0.11875 (9)	0.0244 (2)	
C34	0.42990 (9)	0.87357 (10)	0.13558 (9)	0.0241 (2)	
H34	0.392902	0.880209	0.078243	0.029*	
C34A	0.41068 (9)	0.97731 (10)	0.14211 (9)	0.0241 (2)	
H34A	0.422640	1.028174	0.076711	0.029*	
C35	0.25471 (10)	1.14608 (10)	0.24413 (10)	0.0280 (3)	
C36	0.15875 (11)	1.15125 (13)	0.23216 (13)	0.0391 (3)	
H36	0.140567	1.105368	0.210312	0.047*	
C37	0.08958 (12)	1.22263 (16)	0.25179 (16)	0.0487 (4)	
H37	0.024169	1.225946	0.243151	0.058*	
C38	0.11582 (13)	1.28889 (17)	0.28389 (16)	0.0515 (5)	
C39	0.21008 (12)	1.28380 (15)	0.29891 (14)	0.0456 (4)	
H39	0.227394	1.328634	0.322747	0.055*	
C40	0.27941 (11)	1.21279 (12)	0.27900 (11)	0.0343 (3)	
H40	0.344607	1.209207	0.289095	0.041*	
C41	0.82026 (10)	0.76791 (11)	0.25219 (11)	0.0299 (3)	
C42	0.80948 (10)	0.78885 (12)	0.33545 (11)	0.0331 (3)	
H42	0.788668	0.857143	0.330418	0.040*	
C43	0.82990 (10)	0.70745 (13)	0.42588 (11)	0.0358 (3)	
H43	0.823916	0.720772	0.483053	0.043*	
C44	0.85902 (10)	0.60650 (13)	0.43461 (11)	0.0330 (3)	
C45	0.87021 (10)	0.58859 (12)	0.34965 (11)	0.0326 (3)	
H45	0.891023	0.520392	0.354419	0.039*	
C46	0.85150 (10)	0.66839 (12)	0.25866 (11)	0.0314 (3)	
H46	0.859911	0.655317	0.201374	0.038*	
C47	0.87669 (11)	0.51812 (14)	0.53267 (11)	0.0404 (4)	
H47A	0.815749	0.508052	0.562480	0.061*	
H47B	0.915160	0.532754	0.574652	0.061*	
H47C	0.910679	0.455967	0.524737	0.061*	
C48	0.40092 (9)	0.79273 (10)	0.22781 (9)	0.0248 (2)	
H50	0.300 (2)	0.496 (2)	0.146 (2)	0.076 (8)*	
H100	0.4104 (18)	0.6545 (19)	0.2845 (19)	0.062 (7)*	

### (3aRS,10SR,10aSR)-2-(4-Chlorophenyl)-5-[(4-methylphenyl)sulfonyl]-1-oxo-1,2,3,3a,4,5,10,10a-octahydropyrrolo[3,4-b]carbazole-10-carboxylic acid Atomic displacement parameters (Å^2^)

**Table:**

	*U*^11^	*U*^22^	*U*^33^	*U*^12^	*U*^13^	*U*^23^
Cl1	0.03391 (18)	0.0431 (2)	0.03821 (18)	−0.00323 (14)	0.00171 (14)	−0.02447 (15)
S1	0.03139 (17)	0.02205 (15)	0.02885 (16)	−0.00388 (12)	−0.00107 (12)	−0.00986 (12)
O1	0.0488 (6)	0.0210 (5)	0.0258 (5)	−0.0086 (4)	0.0061 (4)	−0.0055 (4)
O2	0.0370 (5)	0.0244 (5)	0.0314 (5)	−0.0071 (4)	0.0018 (4)	−0.0067 (4)
O3	0.0440 (6)	0.0273 (5)	0.0372 (5)	−0.0038 (4)	−0.0006 (4)	−0.0156 (4)
O4	0.0341 (6)	0.0479 (6)	0.0285 (5)	0.0023 (5)	0.0002 (4)	−0.0126 (5)
O5	0.0360 (6)	0.0575 (7)	0.0258 (5)	−0.0058 (5)	−0.0004 (4)	−0.0155 (5)
C1	0.0301 (6)	0.0234 (6)	0.0228 (6)	−0.0070 (5)	−0.0004 (5)	−0.0063 (5)
N2	0.0292 (5)	0.0203 (5)	0.0214 (5)	−0.0045 (4)	0.0003 (4)	−0.0052 (4)
C3	0.0290 (6)	0.0204 (6)	0.0222 (6)	−0.0045 (5)	−0.0009 (5)	−0.0054 (5)
C3A	0.0276 (6)	0.0217 (6)	0.0221 (6)	−0.0060 (5)	−0.0004 (5)	−0.0056 (5)
C4	0.0310 (6)	0.0226 (6)	0.0237 (6)	−0.0057 (5)	0.0007 (5)	−0.0063 (5)
C4A	0.0271 (6)	0.0243 (6)	0.0261 (6)	−0.0059 (5)	−0.0007 (5)	−0.0101 (5)
N5	0.0293 (6)	0.0255 (5)	0.0264 (5)	−0.0056 (4)	−0.0008 (4)	−0.0104 (4)
C5A	0.0276 (6)	0.0307 (7)	0.0267 (6)	−0.0088 (5)	−0.0001 (5)	−0.0124 (5)
C6	0.0295 (7)	0.0330 (7)	0.0331 (7)	−0.0073 (5)	0.0000 (5)	−0.0166 (6)
C7	0.0325 (7)	0.0436 (8)	0.0317 (7)	−0.0104 (6)	0.0046 (6)	−0.0206 (6)
C8	0.0342 (7)	0.0416 (8)	0.0260 (6)	−0.0120 (6)	0.0026 (5)	−0.0131 (6)
C9	0.0301 (7)	0.0325 (7)	0.0267 (6)	−0.0081 (5)	−0.0001 (5)	−0.0095 (5)
C9A	0.0267 (6)	0.0299 (7)	0.0257 (6)	−0.0080 (5)	−0.0003 (5)	−0.0116 (5)
C9B	0.0265 (6)	0.0262 (6)	0.0252 (6)	−0.0070 (5)	0.0004 (5)	−0.0091 (5)
C10	0.0309 (7)	0.0239 (6)	0.0234 (6)	−0.0077 (5)	0.0029 (5)	−0.0069 (5)
C10A	0.0290 (6)	0.0219 (6)	0.0221 (6)	−0.0068 (5)	0.0006 (5)	−0.0064 (5)
C11	0.0262 (6)	0.0239 (6)	0.0231 (6)	−0.0066 (5)	0.0007 (5)	−0.0076 (5)
C12	0.0331 (7)	0.0252 (6)	0.0239 (6)	−0.0038 (5)	−0.0013 (5)	−0.0062 (5)
C13	0.0332 (7)	0.0247 (6)	0.0312 (7)	−0.0019 (5)	−0.0014 (5)	−0.0091 (5)
C14	0.0268 (6)	0.0329 (7)	0.0311 (7)	−0.0070 (5)	0.0012 (5)	−0.0154 (6)
C15	0.0296 (7)	0.0310 (7)	0.0237 (6)	−0.0063 (5)	−0.0006 (5)	−0.0090 (5)
C16	0.0281 (6)	0.0248 (6)	0.0240 (6)	−0.0038 (5)	−0.0017 (5)	−0.0062 (5)
C17	0.0295 (7)	0.0230 (6)	0.0327 (7)	−0.0011 (5)	−0.0042 (5)	−0.0089 (5)
C18	0.0388 (8)	0.0259 (7)	0.0310 (7)	−0.0025 (6)	−0.0052 (6)	−0.0080 (5)
C19	0.0458 (9)	0.0317 (7)	0.0427 (8)	−0.0016 (6)	−0.0155 (7)	−0.0128 (6)
C20	0.0328 (8)	0.0299 (8)	0.0644 (11)	0.0012 (6)	−0.0154 (7)	−0.0157 (7)
C21	0.0285 (7)	0.0360 (8)	0.0601 (10)	−0.0010 (6)	0.0003 (7)	−0.0166 (7)
C22	0.0308 (7)	0.0324 (7)	0.0411 (8)	0.0000 (6)	−0.0010 (6)	−0.0145 (6)
C23	0.0395 (10)	0.0532 (11)	0.1063 (19)	−0.0052 (8)	−0.0187 (11)	−0.0391 (12)
C24	0.0344 (7)	0.0236 (6)	0.0242 (6)	−0.0055 (5)	0.0007 (5)	−0.0059 (5)
Cl2	0.0339 (4)	0.0531 (6)	0.0738 (7)	0.0020 (4)	0.0042 (5)	−0.0428 (5)
Cl2'	0.0330 (6)	0.0651 (11)	0.0739 (11)	0.0038 (7)	0.0017 (7)	−0.0489 (9)
S2	0.02403 (16)	0.03161 (17)	0.03503 (18)	−0.00549 (12)	0.00296 (13)	−0.01419 (14)
O6	0.0263 (5)	0.0404 (6)	0.0371 (5)	−0.0025 (4)	−0.0049 (4)	−0.0178 (5)
O7	0.0288 (5)	0.0354 (5)	0.0501 (6)	−0.0097 (4)	0.0054 (4)	−0.0191 (5)
O8	0.0281 (5)	0.0412 (6)	0.0383 (5)	−0.0066 (4)	0.0075 (4)	−0.0161 (5)
O9	0.0414 (6)	0.0253 (5)	0.0261 (5)	−0.0091 (4)	0.0064 (4)	−0.0092 (4)
O10	0.0392 (5)	0.0228 (5)	0.0263 (5)	−0.0081 (4)	0.0052 (4)	−0.0073 (4)
C25	0.0274 (6)	0.0231 (6)	0.0212 (6)	−0.0019 (5)	−0.0023 (5)	−0.0035 (5)
N26	0.0238 (5)	0.0238 (5)	0.0266 (5)	−0.0026 (4)	0.0002 (4)	−0.0074 (4)
C27	0.0233 (6)	0.0246 (6)	0.0290 (6)	−0.0041 (5)	0.0003 (5)	−0.0091 (5)
C27A	0.0252 (6)	0.0221 (6)	0.0231 (6)	−0.0039 (5)	−0.0006 (5)	−0.0059 (5)
C28	0.0248 (6)	0.0263 (6)	0.0291 (6)	−0.0045 (5)	−0.0001 (5)	−0.0104 (5)
C28A	0.0252 (6)	0.0235 (6)	0.0222 (6)	−0.0031 (5)	−0.0001 (5)	−0.0053 (5)
N29	0.0260 (5)	0.0275 (5)	0.0263 (5)	−0.0035 (4)	0.0007 (4)	−0.0104 (4)
C29A	0.0317 (7)	0.0235 (6)	0.0200 (5)	−0.0019 (5)	−0.0016 (5)	−0.0051 (5)
C30	0.0329 (7)	0.0300 (7)	0.0232 (6)	−0.0020 (5)	0.0017 (5)	−0.0072 (5)
C31	0.0420 (8)	0.0311 (7)	0.0212 (6)	0.0006 (6)	0.0007 (5)	−0.0089 (5)
C32	0.0440 (8)	0.0300 (7)	0.0226 (6)	−0.0037 (6)	−0.0038 (6)	−0.0102 (5)
C33	0.0352 (7)	0.0261 (6)	0.0220 (6)	−0.0037 (5)	−0.0045 (5)	−0.0062 (5)
C33A	0.0310 (6)	0.0222 (6)	0.0176 (5)	−0.0020 (5)	−0.0022 (5)	−0.0026 (5)
C33B	0.0282 (6)	0.0208 (6)	0.0190 (5)	−0.0030 (5)	−0.0020 (5)	−0.0040 (4)
C34	0.0256 (6)	0.0230 (6)	0.0202 (5)	−0.0035 (5)	−0.0019 (4)	−0.0058 (5)
C34A	0.0249 (6)	0.0212 (6)	0.0201 (5)	−0.0024 (5)	−0.0018 (4)	−0.0033 (4)
C35	0.0283 (6)	0.0250 (6)	0.0253 (6)	−0.0021 (5)	0.0022 (5)	−0.0070 (5)
C36	0.0302 (7)	0.0406 (8)	0.0526 (9)	−0.0080 (6)	0.0048 (6)	−0.0255 (7)
C37	0.0268 (7)	0.0615 (11)	0.0684 (12)	−0.0043 (7)	0.0015 (7)	−0.0400 (10)
C38	0.0318 (8)	0.0665 (12)	0.0697 (12)	0.0036 (8)	−0.0005 (8)	−0.0488 (11)
C39	0.0352 (8)	0.0574 (10)	0.0559 (10)	−0.0006 (7)	−0.0021 (7)	−0.0397 (9)
C40	0.0287 (7)	0.0398 (8)	0.0358 (7)	−0.0016 (6)	−0.0021 (6)	−0.0196 (6)
C41	0.0226 (6)	0.0357 (7)	0.0331 (7)	−0.0050 (5)	−0.0003 (5)	−0.0163 (6)
C42	0.0249 (6)	0.0402 (8)	0.0407 (8)	−0.0037 (6)	−0.0004 (6)	−0.0244 (6)
C43	0.0255 (7)	0.0536 (9)	0.0356 (7)	−0.0060 (6)	−0.0008 (5)	−0.0267 (7)
C44	0.0200 (6)	0.0473 (8)	0.0324 (7)	−0.0036 (6)	−0.0026 (5)	−0.0184 (6)
C45	0.0260 (6)	0.0376 (8)	0.0344 (7)	−0.0018 (6)	−0.0020 (5)	−0.0173 (6)
C46	0.0274 (6)	0.0380 (7)	0.0314 (7)	−0.0034 (5)	−0.0004 (5)	−0.0188 (6)
C47	0.0295 (7)	0.0557 (10)	0.0313 (7)	−0.0039 (7)	−0.0036 (6)	−0.0155 (7)
C48	0.0246 (6)	0.0245 (6)	0.0230 (6)	−0.0048 (5)	−0.0028 (5)	−0.0073 (5)

### (3aRS,10SR,10aSR)-2-(4-Chlorophenyl)-5-[(4-methylphenyl)sulfonyl]-1-oxo-1,2,3,3a,4,5,10,10a-octahydropyrrolo[3,4-b]carbazole-10-carboxylic acid Geometric parameters (Å, º)

**Table:**

Cl1—C14	1.7403 (14)	Cl2'—C38	1.756 (2)
S1—O2	1.4254 (11)	S2—O8	1.4261 (11)
S1—O3	1.4280 (11)	S2—O7	1.4280 (11)
S1—N5	1.6752 (12)	S2—N29	1.6794 (12)
S1—C17	1.7587 (15)	S2—C41	1.7587 (15)
O1—C1	1.2334 (17)	O6—C25	1.2166 (17)
O4—C24	1.2076 (18)	O9—C48	1.2072 (17)
O5—C24	1.3198 (17)	O10—C48	1.3265 (17)
O5—H50	1.00 (3)	O10—H100	0.90 (3)
C1—N2	1.3629 (17)	C25—N26	1.3806 (18)
C1—C10A	1.4985 (19)	C25—C34A	1.5093 (18)
N2—C11	1.4175 (17)	N26—C35	1.4142 (17)
N2—C3	1.4829 (16)	N26—C27	1.4846 (17)
C3—C3A	1.5283 (18)	C27—C27A	1.5281 (18)
C3—H3A	0.9900	C27—H27A	0.9900
C3—H3B	0.9900	C27—H27B	0.9900
C3A—C4	1.5228 (18)	C27A—C28	1.5244 (18)
C3A—C10A	1.5279 (17)	C27A—C34A	1.5247 (18)
C3A—H3C	1.0000	C27A—H27C	1.0000
C4—C4A	1.5064 (18)	C28—C28A	1.5056 (19)
C4—H4A	0.9900	C28—H28A	0.9900
C4—H4B	0.9900	C28—H28B	0.9900
C4A—C9B	1.3645 (18)	C28A—C33B	1.3608 (19)
C4A—N5	1.4236 (17)	C28A—N29	1.4209 (17)
N5—C5A	1.4255 (17)	N29—C29A	1.4221 (18)
C5A—C6	1.392 (2)	C29A—C30	1.397 (2)
C5A—C9A	1.404 (2)	C29A—C33A	1.405 (2)
C6—C7	1.387 (2)	C30—C31	1.387 (2)
C6—H6	0.9500	C30—H30	0.9500
C7—C8	1.400 (2)	C31—C32	1.401 (2)
C7—H7	0.9500	C31—H31	0.9500
C8—C9	1.385 (2)	C32—C33	1.389 (2)
C8—H8	0.9500	C32—H32	0.9500
C9—C9A	1.4010 (19)	C33—C33A	1.4021 (19)
C9—H9	0.9500	C33—H33	0.9500
C9A—C9B	1.4389 (19)	C33A—C33B	1.4445 (18)
C9B—C10	1.5119 (19)	C33B—C34	1.5181 (18)
C10—C10A	1.5197 (18)	C34—C34A	1.5255 (18)
C10—C24	1.525 (2)	C34—C48	1.5292 (17)
C10—H10	1.0000	C34—H34	1.0000
C10A—H10A	1.0000	C34A—H34A	1.0000
C11—C16	1.3948 (18)	C35—C36	1.391 (2)
C11—C12	1.3979 (19)	C35—C40	1.401 (2)
C12—C13	1.387 (2)	C36—C37	1.383 (2)
C12—H12	0.9500	C36—H36	0.9500
C13—C14	1.387 (2)	C37—C38	1.377 (3)
C13—H13	0.9500	C37—H37	0.9500
C14—C15	1.382 (2)	C38—C39	1.375 (2)
C15—C16	1.389 (2)	C39—C40	1.383 (2)
C15—H15	0.9500	C39—H39	0.9500
C16—H16	0.9500	C40—H40	0.9500
C17—C18	1.392 (2)	C41—C46	1.390 (2)
C17—C22	1.394 (2)	C41—C42	1.396 (2)
C18—C19	1.387 (2)	C42—C43	1.390 (2)
C18—H18	0.9500	C42—H42	0.9500
C19—C20	1.394 (3)	C43—C44	1.396 (2)
C19—H19	0.9500	C43—H43	0.9500
C20—C21	1.393 (3)	C44—C45	1.397 (2)
C20—C23	1.511 (2)	C44—C47	1.504 (2)
C21—C22	1.383 (2)	C45—C46	1.384 (2)
C21—H21	0.9500	C45—H45	0.9500
C22—H22	0.9500	C46—H46	0.9500
C23—H23A	0.9800	C47—H47A	0.9800
C23—H23B	0.9800	C47—H47B	0.9800
C23—H23C	0.9800	C47—H47C	0.9800
Cl2—C38	1.784 (2)		
			
O2—S1—O3	120.00 (7)	O8—S2—N29	106.01 (6)
O2—S1—N5	106.74 (6)	O7—S2—N29	106.53 (6)
O3—S1—N5	105.72 (6)	O8—S2—C41	108.50 (7)
O2—S1—C17	108.78 (7)	O7—S2—C41	109.43 (7)
O3—S1—C17	109.27 (7)	N29—S2—C41	104.75 (6)
N5—S1—C17	105.33 (6)	C48—O10—H100	109.4 (16)
C24—O5—H50	110.8 (16)	O6—C25—N26	127.04 (13)
O1—C1—N2	124.76 (13)	O6—C25—C34A	125.90 (13)
O1—C1—C10A	127.19 (12)	N26—C25—C34A	106.95 (11)
N2—C1—C10A	107.81 (11)	C25—N26—C35	125.73 (12)
C1—N2—C11	125.35 (11)	C25—N26—C27	112.10 (11)
C1—N2—C3	111.44 (11)	C35—N26—C27	121.14 (11)
C11—N2—C3	122.16 (10)	N26—C27—C27A	102.21 (10)
N2—C3—C3A	102.22 (10)	N26—C27—H27A	111.3
N2—C3—H3A	111.3	C27A—C27—H27A	111.3
C3A—C3—H3A	111.3	N26—C27—H27B	111.3
N2—C3—H3B	111.3	C27A—C27—H27B	111.3
C3A—C3—H3B	111.3	H27A—C27—H27B	109.2
H3A—C3—H3B	109.2	C28—C27A—C34A	111.38 (11)
C4—C3A—C10A	111.01 (11)	C28—C27A—C27	118.13 (11)
C4—C3A—C3	117.45 (11)	C34A—C27A—C27	102.46 (10)
C10A—C3A—C3	101.34 (10)	C28—C27A—H27C	108.1
C4—C3A—H3C	108.9	C34A—C27A—H27C	108.1
C10A—C3A—H3C	108.9	C27—C27A—H27C	108.1
C3—C3A—H3C	108.9	C28A—C28—C27A	107.50 (11)
C4A—C4—C3A	107.55 (10)	C28A—C28—H28A	110.2
C4A—C4—H4A	110.2	C27A—C28—H28A	110.2
C3A—C4—H4A	110.2	C28A—C28—H28B	110.2
C4A—C4—H4B	110.2	C27A—C28—H28B	110.2
C3A—C4—H4B	110.2	H28A—C28—H28B	108.5
H4A—C4—H4B	108.5	C33B—C28A—N29	108.71 (12)
C9B—C4A—N5	108.62 (12)	C33B—C28A—C28	126.78 (12)
C9B—C4A—C4	126.54 (12)	N29—C28A—C28	124.25 (12)
N5—C4A—C4	124.84 (11)	C28A—N29—C29A	108.06 (11)
C4A—N5—C5A	107.78 (11)	C28A—N29—S2	124.39 (10)
C4A—N5—S1	123.90 (9)	C29A—N29—S2	124.01 (10)
C5A—N5—S1	123.21 (10)	C30—C29A—C33A	121.85 (13)
C6—C5A—C9A	121.80 (13)	C30—C29A—N29	130.89 (13)
C6—C5A—N5	130.99 (13)	C33A—C29A—N29	107.13 (12)
C9A—C5A—N5	107.19 (12)	C31—C30—C29A	117.37 (14)
C7—C6—C5A	117.33 (14)	C31—C30—H30	121.3
C7—C6—H6	121.3	C29A—C30—H30	121.3
C5A—C6—H6	121.3	C30—C31—C32	121.62 (14)
C6—C7—C8	121.70 (14)	C30—C31—H31	119.2
C6—C7—H7	119.2	C32—C31—H31	119.2
C8—C7—H7	119.2	C33—C32—C31	120.80 (14)
C9—C8—C7	120.77 (13)	C33—C32—H32	119.6
C9—C8—H8	119.6	C31—C32—H32	119.6
C7—C8—H8	119.6	C32—C33—C33A	118.57 (14)
C8—C9—C9A	118.49 (14)	C32—C33—H33	120.7
C8—C9—H9	120.8	C33A—C33—H33	120.7
C9A—C9—H9	120.8	C33—C33A—C29A	119.78 (13)
C9—C9A—C5A	119.91 (13)	C33—C33A—C33B	132.55 (13)
C9—C9A—C9B	132.35 (13)	C29A—C33A—C33B	107.59 (12)
C5A—C9A—C9B	107.73 (12)	C28A—C33B—C33A	108.48 (12)
C4A—C9B—C9A	108.58 (12)	C28A—C33B—C34	123.38 (12)
C4A—C9B—C10	123.73 (12)	C33A—C33B—C34	127.75 (12)
C9A—C9B—C10	127.45 (12)	C33B—C34—C34A	106.01 (10)
C9B—C10—C10A	105.82 (11)	C33B—C34—C48	112.17 (10)
C9B—C10—C24	113.37 (11)	C34A—C34—C48	111.28 (11)
C10A—C10—C24	112.18 (11)	C33B—C34—H34	109.1
C9B—C10—H10	108.4	C34A—C34—H34	109.1
C10A—C10—H10	108.4	C48—C34—H34	109.1
C24—C10—H10	108.4	C25—C34A—C27A	103.75 (11)
C1—C10A—C10	119.90 (11)	C25—C34A—C34	119.18 (11)
C1—C10A—C3A	103.71 (10)	C27A—C34A—C34	112.02 (10)
C10—C10A—C3A	112.64 (11)	C25—C34A—H34A	107.1
C1—C10A—H10A	106.6	C27A—C34A—H34A	107.1
C10—C10A—H10A	106.6	C34—C34A—H34A	107.1
C3A—C10A—H10A	106.6	C36—C35—C40	118.46 (13)
C16—C11—C12	119.41 (13)	C36—C35—N26	121.83 (13)
C16—C11—N2	119.22 (12)	C40—C35—N26	119.70 (13)
C12—C11—N2	121.36 (12)	C37—C36—C35	120.56 (15)
C13—C12—C11	119.98 (12)	C37—C36—H36	119.7
C13—C12—H12	120.0	C35—C36—H36	119.7
C11—C12—H12	120.0	C38—C37—C36	119.80 (16)
C12—C13—C14	119.88 (13)	C38—C37—H37	120.1
C12—C13—H13	120.1	C36—C37—H37	120.1
C14—C13—H13	120.1	C39—C38—C37	120.97 (16)
C15—C14—C13	120.75 (13)	C39—C38—Cl2'	121.78 (15)
C15—C14—Cl1	119.81 (11)	C37—C38—Cl2'	115.33 (15)
C13—C14—Cl1	119.44 (11)	C39—C38—Cl2	118.05 (15)
C14—C15—C16	119.52 (13)	C37—C38—Cl2	120.42 (14)
C14—C15—H15	120.2	C38—C39—C40	119.36 (16)
C16—C15—H15	120.2	C38—C39—H39	120.3
C15—C16—C11	120.46 (12)	C40—C39—H39	120.3
C15—C16—H16	119.8	C39—C40—C35	120.82 (14)
C11—C16—H16	119.8	C39—C40—H40	119.6
C18—C17—C22	121.00 (14)	C35—C40—H40	119.6
C18—C17—S1	119.21 (12)	C46—C41—C42	121.27 (14)
C22—C17—S1	119.79 (12)	C46—C41—S2	118.52 (11)
C19—C18—C17	119.08 (15)	C42—C41—S2	120.21 (12)
C19—C18—H18	120.5	C43—C42—C41	118.44 (14)
C17—C18—H18	120.5	C43—C42—H42	120.8
C18—C19—C20	121.03 (16)	C41—C42—H42	120.8
C18—C19—H19	119.5	C42—C43—C44	121.49 (14)
C20—C19—H19	119.5	C42—C43—H43	119.3
C21—C20—C19	118.63 (15)	C44—C43—H43	119.3
C21—C20—C23	120.63 (19)	C43—C44—C45	118.45 (14)
C19—C20—C23	120.70 (19)	C43—C44—C47	121.45 (14)
C22—C21—C20	121.50 (16)	C45—C44—C47	120.10 (15)
C22—C21—H21	119.3	C46—C45—C44	121.19 (14)
C20—C21—H21	119.3	C46—C45—H45	119.4
C21—C22—C17	118.75 (16)	C44—C45—H45	119.4
C21—C22—H22	120.6	C45—C46—C41	119.13 (13)
C17—C22—H22	120.6	C45—C46—H46	120.4
C20—C23—H23A	109.5	C41—C46—H46	120.4
C20—C23—H23B	109.5	C44—C47—H47A	109.5
H23A—C23—H23B	109.5	C44—C47—H47B	109.5
C20—C23—H23C	109.5	H47A—C47—H47B	109.5
H23A—C23—H23C	109.5	C44—C47—H47C	109.5
H23B—C23—H23C	109.5	H47A—C47—H47C	109.5
O4—C24—O5	123.86 (14)	H47B—C47—H47C	109.5
O4—C24—C10	123.21 (13)	O9—C48—O10	124.10 (12)
O5—C24—C10	112.93 (12)	O9—C48—C34	123.51 (12)
O8—S2—O7	120.47 (7)	O10—C48—C34	112.36 (11)
			
O1—C1—N2—C11	−5.2 (2)	C34A—C25—N26—C35	167.00 (11)
C10A—C1—N2—C11	169.43 (12)	O6—C25—N26—C27	−177.62 (13)
O1—C1—N2—C3	−173.64 (13)	C34A—C25—N26—C27	−1.38 (14)
C10A—C1—N2—C3	0.99 (15)	C25—N26—C27—C27A	−20.23 (13)
C1—N2—C3—C3A	−23.09 (14)	C35—N26—C27—C27A	170.79 (11)
C11—N2—C3—C3A	168.05 (11)	N26—C27—C27A—C28	155.45 (11)
N2—C3—C3A—C4	155.48 (11)	N26—C27—C27A—C34A	32.67 (12)
N2—C3—C3A—C10A	34.40 (12)	C34A—C27A—C28—C28A	−42.74 (14)
C10A—C3A—C4—C4A	−42.50 (14)	C27—C27A—C28—C28A	−160.91 (11)
C3—C3A—C4—C4A	−158.40 (11)	C27A—C28—C28A—C33B	9.90 (18)
C3A—C4—C4A—C9B	9.26 (18)	C27A—C28—C28A—N29	−176.59 (11)
C3A—C4—C4A—N5	−170.63 (12)	C33B—C28A—N29—C29A	−1.82 (14)
C9B—C4A—N5—C5A	2.99 (15)	C28—C28A—N29—C29A	−176.33 (12)
C4—C4A—N5—C5A	−177.11 (12)	C33B—C28A—N29—S2	−161.24 (9)
C9B—C4A—N5—S1	158.27 (10)	C28—C28A—N29—S2	24.25 (17)
C4—C4A—N5—S1	−21.82 (18)	O8—S2—N29—C28A	−169.58 (10)
O2—S1—N5—C4A	39.01 (12)	O7—S2—N29—C28A	−40.12 (12)
O3—S1—N5—C4A	167.84 (11)	C41—S2—N29—C28A	75.78 (12)
C17—S1—N5—C4A	−76.52 (12)	O8—S2—N29—C29A	34.19 (12)
O2—S1—N5—C5A	−169.40 (10)	O7—S2—N29—C29A	163.65 (11)
O3—S1—N5—C5A	−40.57 (12)	C41—S2—N29—C29A	−80.44 (12)
C17—S1—N5—C5A	75.06 (12)	C28A—N29—C29A—C30	177.30 (13)
C4A—N5—C5A—C6	177.77 (14)	S2—N29—C29A—C30	−23.2 (2)
S1—N5—C5A—C6	22.3 (2)	C28A—N29—C29A—C33A	1.56 (14)
C4A—N5—C5A—C9A	−3.24 (14)	S2—N29—C29A—C33A	161.07 (9)
S1—N5—C5A—C9A	−158.74 (10)	C33A—C29A—C30—C31	−0.62 (19)
C9A—C5A—C6—C7	−0.8 (2)	N29—C29A—C30—C31	−175.83 (13)
N5—C5A—C6—C7	178.05 (13)	C29A—C30—C31—C32	0.9 (2)
C5A—C6—C7—C8	0.6 (2)	C30—C31—C32—C33	−0.8 (2)
C6—C7—C8—C9	−0.1 (2)	C31—C32—C33—C33A	0.2 (2)
C7—C8—C9—C9A	−0.3 (2)	C32—C33—C33A—C29A	0.05 (19)
C8—C9—C9A—C5A	0.1 (2)	C32—C33—C33A—C33B	176.30 (13)
C8—C9—C9A—C9B	178.90 (14)	C30—C29A—C33A—C33	0.15 (19)
C6—C5A—C9A—C9	0.5 (2)	N29—C29A—C33A—C33	176.36 (11)
N5—C5A—C9A—C9	−178.64 (12)	C30—C29A—C33A—C33B	−176.96 (12)
C6—C5A—C9A—C9B	−178.59 (12)	N29—C29A—C33A—C33B	−0.75 (14)
N5—C5A—C9A—C9B	2.31 (15)	N29—C28A—C33B—C33A	1.34 (14)
N5—C4A—C9B—C9A	−1.55 (15)	C28—C28A—C33B—C33A	175.68 (12)
C4—C4A—C9B—C9A	178.55 (12)	N29—C28A—C33B—C34	−172.05 (11)
N5—C4A—C9B—C10	−176.20 (12)	C28—C28A—C33B—C34	2.3 (2)
C4—C4A—C9B—C10	3.9 (2)	C33—C33A—C33B—C28A	−176.96 (13)
C9—C9A—C9B—C4A	−179.38 (14)	C29A—C33A—C33B—C28A	−0.37 (14)
C5A—C9A—C9B—C4A	−0.49 (15)	C33—C33A—C33B—C34	−3.9 (2)
C9—C9A—C9B—C10	−5.0 (2)	C29A—C33A—C33B—C34	172.65 (11)
C5A—C9A—C9B—C10	173.91 (13)	C28A—C33B—C34—C34A	18.25 (16)
C4A—C9B—C10—C10A	16.76 (17)	C33A—C33B—C34—C34A	−153.81 (12)
C9A—C9B—C10—C10A	−156.85 (13)	C28A—C33B—C34—C48	−103.40 (14)
C4A—C9B—C10—C24	−106.60 (15)	C33A—C33B—C34—C48	84.55 (15)
C9A—C9B—C10—C24	79.79 (17)	O6—C25—C34A—C27A	−161.10 (13)
O1—C1—C10A—C10	−37.2 (2)	N26—C25—C34A—C27A	22.61 (13)
N2—C1—C10A—C10	148.33 (12)	O6—C25—C34A—C34	−35.73 (19)
O1—C1—C10A—C3A	−163.88 (14)	N26—C25—C34A—C34	147.98 (11)
N2—C1—C10A—C3A	21.66 (14)	C28—C27A—C34A—C25	−161.33 (10)
C9B—C10—C10A—C1	−173.64 (11)	C27—C27A—C34A—C25	−34.10 (12)
C24—C10—C10A—C1	−49.53 (16)	C28—C27A—C34A—C34	68.84 (13)
C9B—C10—C10A—C3A	−51.22 (14)	C27—C27A—C34A—C34	−163.93 (10)
C24—C10—C10A—C3A	72.88 (14)	C33B—C34—C34A—C25	−173.04 (10)
C4—C3A—C10A—C1	−159.93 (11)	C48—C34—C34A—C25	−50.82 (15)
C3—C3A—C10A—C1	−34.43 (12)	C33B—C34—C34A—C27A	−51.73 (13)
C4—C3A—C10A—C10	68.95 (14)	C48—C34—C34A—C27A	70.48 (13)
C3—C3A—C10A—C10	−165.55 (11)	C25—N26—C35—C36	11.1 (2)
C1—N2—C11—C16	−149.36 (13)	C27—N26—C35—C36	178.56 (13)
C3—N2—C11—C16	17.91 (18)	C25—N26—C35—C40	−167.87 (13)
C1—N2—C11—C12	31.9 (2)	C27—N26—C35—C40	−0.46 (19)
C3—N2—C11—C12	−160.87 (12)	C40—C35—C36—C37	1.7 (2)
C16—C11—C12—C13	−0.3 (2)	N26—C35—C36—C37	−177.28 (16)
N2—C11—C12—C13	178.50 (13)	C35—C36—C37—C38	−0.3 (3)
C11—C12—C13—C14	−0.7 (2)	C36—C37—C38—C39	−1.5 (3)
C12—C13—C14—C15	1.3 (2)	C36—C37—C38—Cl2'	−165.97 (18)
C12—C13—C14—Cl1	−178.94 (11)	C36—C37—C38—Cl2	169.80 (17)
C13—C14—C15—C16	−0.8 (2)	C37—C38—C39—C40	1.7 (3)
Cl1—C14—C15—C16	179.41 (11)	Cl2'—C38—C39—C40	165.21 (18)
C14—C15—C16—C11	−0.2 (2)	Cl2—C38—C39—C40	−169.76 (16)
C12—C11—C16—C15	0.8 (2)	C38—C39—C40—C35	−0.2 (3)
N2—C11—C16—C15	−178.05 (12)	C36—C35—C40—C39	−1.5 (2)
O2—S1—C17—C18	−17.25 (13)	N26—C35—C40—C39	177.55 (15)
O3—S1—C17—C18	−149.96 (11)	O8—S2—C41—C46	−37.76 (13)
N5—S1—C17—C18	96.88 (12)	O7—S2—C41—C46	−171.00 (11)
O2—S1—C17—C22	162.60 (11)	N29—S2—C41—C46	75.12 (12)
O3—S1—C17—C22	29.89 (13)	O8—S2—C41—C42	142.59 (12)
N5—S1—C17—C22	−83.27 (12)	O7—S2—C41—C42	9.34 (14)
C22—C17—C18—C19	0.5 (2)	N29—S2—C41—C42	−104.53 (12)
S1—C17—C18—C19	−179.63 (11)	C46—C41—C42—C43	−0.6 (2)
C17—C18—C19—C20	0.1 (2)	S2—C41—C42—C43	179.04 (11)
C18—C19—C20—C21	−0.9 (2)	C41—C42—C43—C44	−1.1 (2)
C18—C19—C20—C23	177.15 (16)	C42—C43—C44—C45	1.9 (2)
C19—C20—C21—C22	0.9 (2)	C42—C43—C44—C47	−177.09 (14)
C23—C20—C21—C22	−177.09 (16)	C43—C44—C45—C46	−1.1 (2)
C20—C21—C22—C17	−0.3 (2)	C47—C44—C45—C46	177.95 (14)
C18—C17—C22—C21	−0.5 (2)	C44—C45—C46—C41	−0.6 (2)
S1—C17—C22—C21	179.69 (12)	C42—C41—C46—C45	1.4 (2)
C9B—C10—C24—O4	121.86 (15)	S2—C41—C46—C45	−178.23 (11)
C10A—C10—C24—O4	2.06 (19)	C33B—C34—C48—O9	122.69 (14)
C9B—C10—C24—O5	−58.28 (16)	C34A—C34—C48—O9	4.12 (18)
C10A—C10—C24—O5	−178.08 (12)	C33B—C34—C48—O10	−55.39 (14)
O6—C25—N26—C35	−9.2 (2)	C34A—C34—C48—O10	−173.97 (11)

### (3aRS,10SR,10aSR)-2-(4-Chlorophenyl)-5-[(4-methylphenyl)sulfonyl]-1-oxo-1,2,3,3a,4,5,10,10a-octahydropyrrolo[3,4-b]carbazole-10-carboxylic acid Hydrogen-bond geometry (Å, º)

*Cg*2, *Cg*4, *Cg*6, *Cg*11 and *Cg*12 are the centroids of the C28*A*/N29/C29*A*/C33*A*/C33*B*, C29*A*/C30–C33/C33*A*, C41–C46, C11–C16 and C17–C22 rings, respectively.

**Table:**

*D*—H···*A*	*D*—H	H···*A*	*D*···*A*	*D*—H···*A*
O10—H100···O1	0.90 (3)	1.74 (3)	2.6219 (14)	166 (2)
C27—H27*A*···O2^i^	0.99	2.48	3.3125 (18)	141
C39—H39···O4^ii^	0.95	2.50	3.380 (3)	155
C42—H42···O3^ii^	0.95	2.49	3.324 (2)	147
C45—H45···Cl2^iii^	0.95	2.80	3.599 (2)	143
C47—H47*A*···O4^i^	0.98	2.48	3.338 (2)	146
C3—H3*A*···*Cg*6^i^	0.99	2.86	3.7276 (16)	147
C13—H13···*Cg*12^i^	0.95	2.60	3.5017 (18)	158
C15—H15···*Cg*4^i^	0.95	2.60	3.4274 (16)	145
C34*A*—H34*A*···*Cg*2^iv^	1.00	2.58	3.5252 (14)	157
C45—H45···*Cg*11^i^	0.95	2.98	3.3309 (17)	103

Symmetry codes: (i) −*x*+1, −*y*+1, −*z*+1; (ii) *x*, *y*+1, *z*; (iii) *x*+1, *y*−1, *z*; (iv) −*x*+1, −*y*+2, −*z*.
