# Supplementary material for: Synthesis and structure of (3aRS,10SR,10aSR)-2-(4-chloro­phen­yl)-5-[(4-methyl­phen­yl)sulfon­yl]-1-oxo-1,2,3,3a,4,5,10,10a-octa­hydro­pyrrolo­[3,4-b]carbazole-10-carb­oxy­lic acid with an unknown solvent
Source: Acta Crystallogr E Crystallogr Commun. 2026 Apr 17;82(Pt 5):480–4. doi: 10.1107/S2056989026003762 (PMC13148214; doi:10.1107/S2056989026003762)
Supplement: Supplementary file 3 [file e-82-00480-sup3.docx]

**Supplementary materials**

| **Synthesis and structure of (3a*RS*,10*SR*,10a*SR*)-2-(4-chlorophenyl)-5-[(4-methylphenyl) sulfonyl]-1-oxo-1,2,3,3a,4,5,10,10a-octahydropyrrolo[3,4-*b*] carbazole-10-carboxylic acid with an unknown solvent** |
| --- |

| **Elizaveta D. Yakovleva,^a^ Elena A. Sorokina,^a^ Victor N. Khrustalev,^b^ Mehmet Akkurt,^c^ Khudayar I. Hasanov,^d^ Narmina A. Guliyeva,^e^ Nurlana D. Sadikhova^f^ and Menberu Mengesha Woldemariam^g^***  **^a^**RUDN University, 6 Miklukho-Maklaya St., Moscow 117198, Russian Federation, **^b^**RUDN University, 6 Miklukho-Maklaya St., Moscow 117198, Russian Federation, and, Zelinsky Institute of Organic Chemistry of RAS, Leninsky Prospect 47, 119991 Moscow, Russian Federation, **^c^**Department of Physics, Faculty of Sciences, Erciyes University, 38039 Kayseri, Turkey, **^d^**Azerbaijan Medical University, Scientific Research Centre (SRC), A. Kasumzade St. 14, AZ 1022, Baku, Azerbaijan, **^e^**Department of Chemical Engineering, Baku Engineering University, Khirdalan, Hasan Aliyev str. 120, AZ0101 Absheron, Azerbaijan, **^f^**Organic Chemistry Department, Baku State University, Z. Khalilov str. 23, AZ 1148, Baku, Azerbaijan, and **^g^**Department of Physics, Jimma University, Jimma, Ethiopia  [Correspondence e-mail: **menberu.mengesha@ju.edu.et**](mailto:Correspondence%20e-mail:%20menberu.mengesha@ju.edu.et)  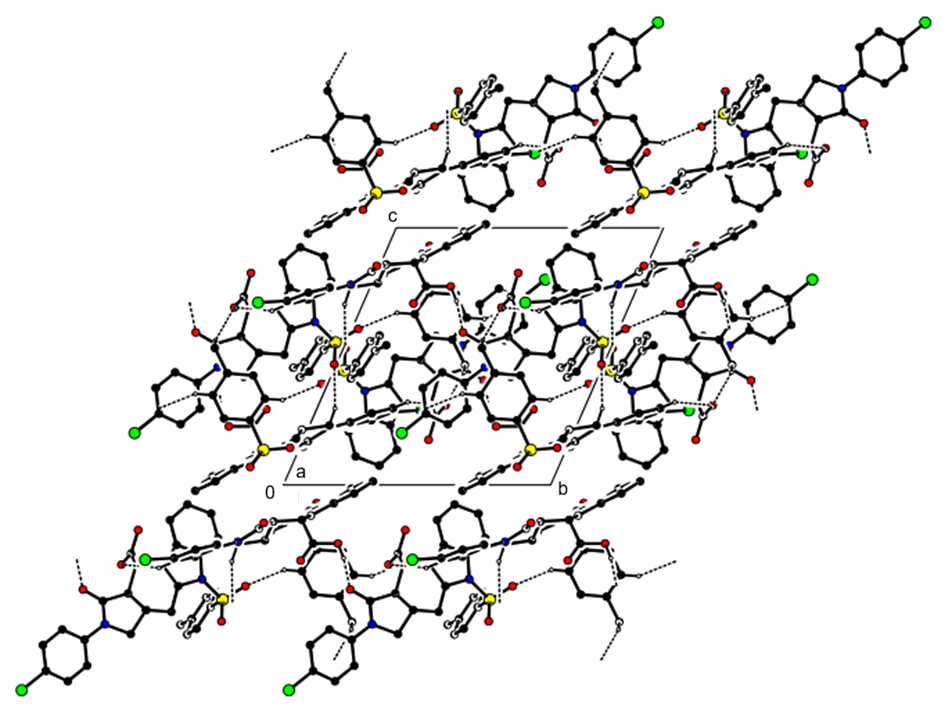  [**Figure S1**.](readonly) The packing of (**I**), viewed down the *a*-axis direction, showing O—H···O, C—H···O and C—H···Cl hydrogen bonds; the major component of the disorder and H-atoms not involved in hydrogen bonds have been omitted for clarity.  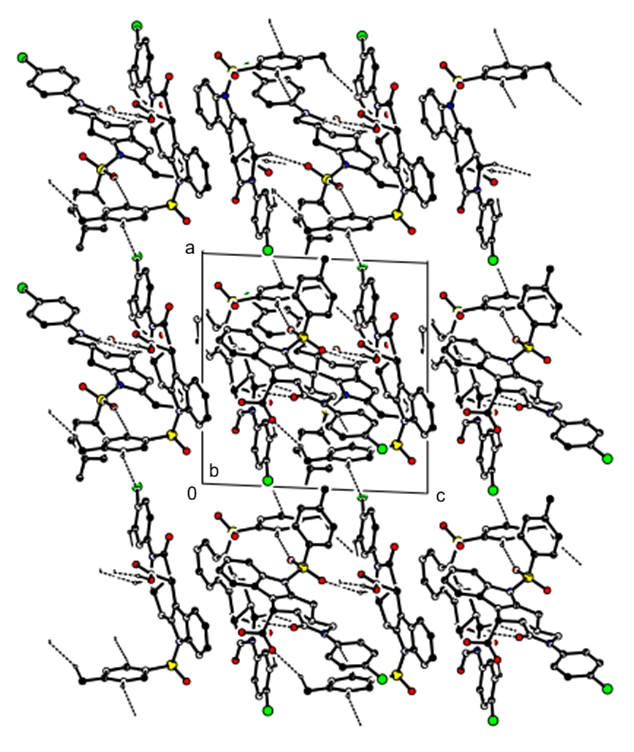  [**Figure S2**.](readonly) The packing of (**I**), showing the O—H···O, C—H···O and C—H···Cl interactions along the *b*-axis direction as dashed lines.  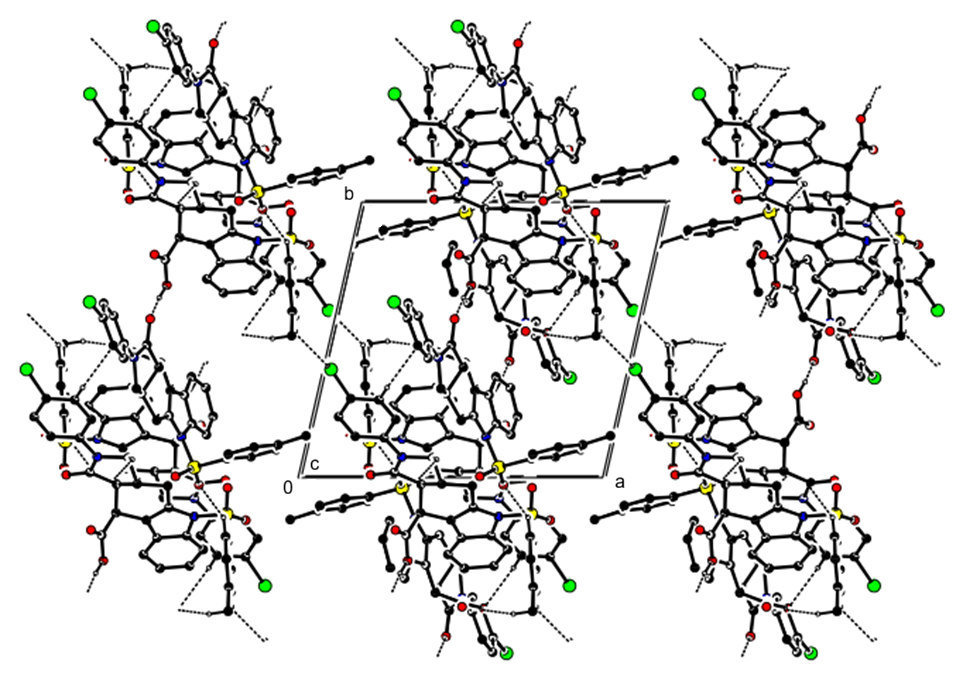  [**Figure S3**.](readonly) The packing of (**I**), showing the O—H···O, C—H···O and C—H···Cl interactions along the *c*-axis direction as dashed lines.  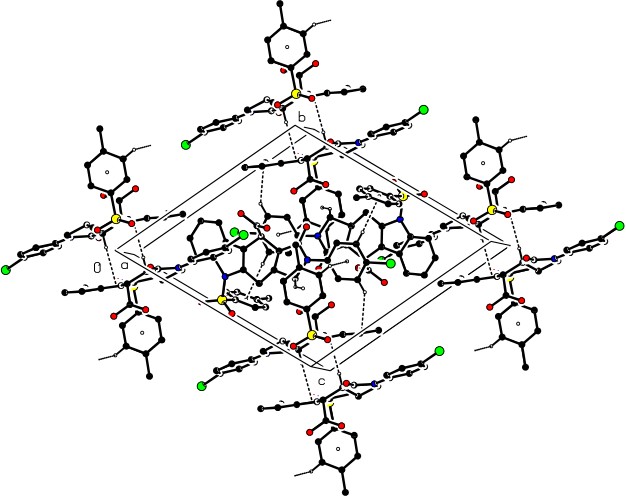  [**Figure S4**.](readonly) A view of the C—H···π interactions of (**I**) viewed along the *a*-axis.  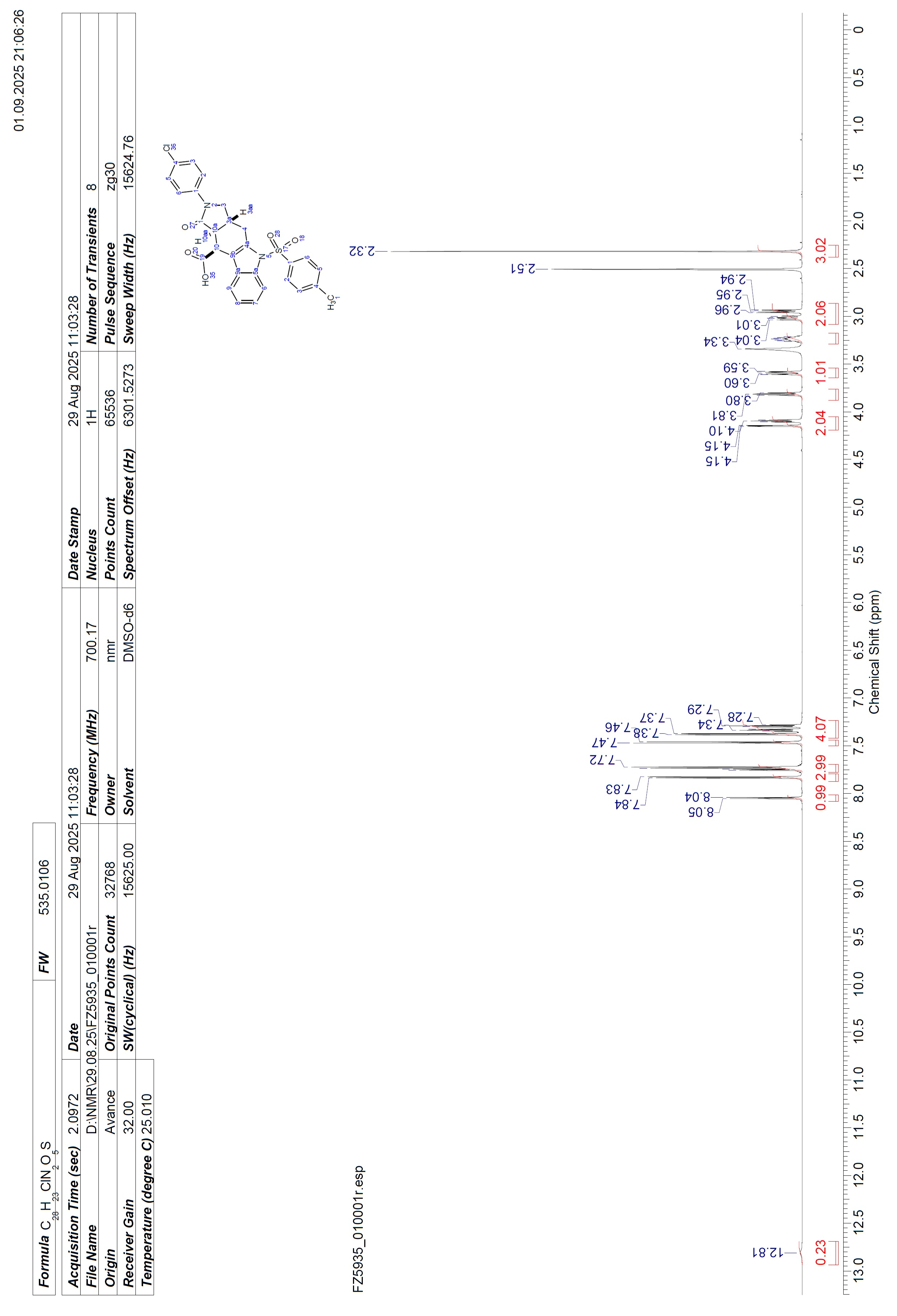  **Figure S5.** ^1^H-NMR spectrum of (**I**).  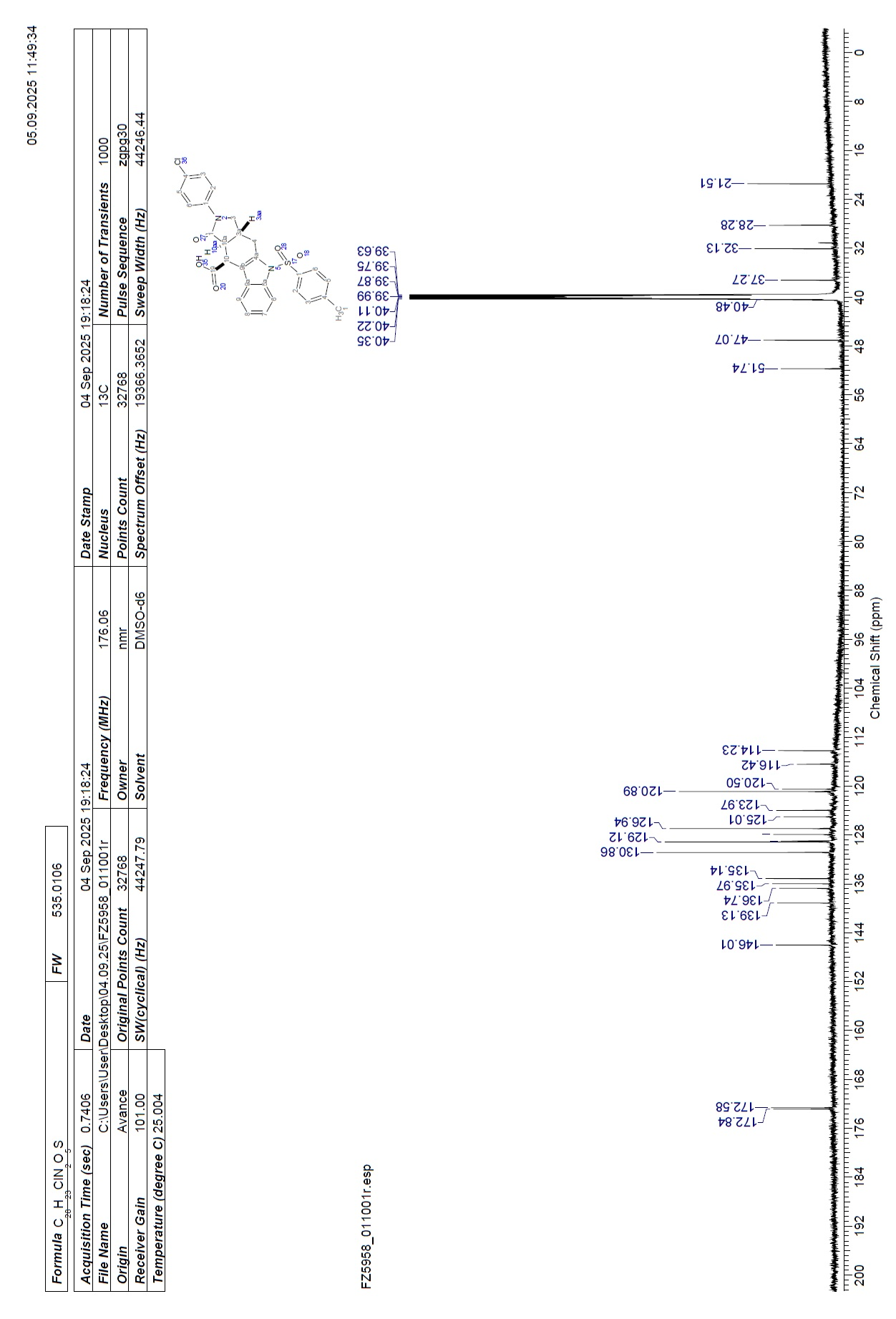  **Figure S6.** ^13^C-NMR spectrum of (**I**). |
| --- |
